# Construction Notes

**Published:** 1895-08-17

**Authors:** 


### CONSTRUCTION NOTES.

**New Wing to the Kendray Infectious Diseases Hospital at Barnsley.**

A new wing has now been formally opened at the above hospital, which extension will be known as "The Lambert Wards," having been added by means of a bequest of £2,000 from the late Mrs. Lambert, a former Barnsley lady, who gave the hospital in the memory of her parents. The original building has proved of great value to the town and surrounding district, but the accommodation was of too limited a nature. Architecturally the new erection is in character with the existing hospital, with which it is connected by a corridor leading into the administration block. The building consists of male and female wards, T shaped, each 61 by 18 feet. There are two special wards for the treatment of exceptional cases. The nurses' day-room is so constructed that a full view is presented of both the wards. This room is fitted with cooking apparatus. The whole of the interior of this extension work presents a bright and attractive appearance, and the rooms are heated with low pressure hot-water pipes. The wards have accommodation for 16 adult patients. The sewerage and drainage is on the most modern and complete scale, and the ventilation is good. The works have been carried out from the plans prepared by Mr. J. H. Taylor, borough surveyor, at a cost of £1,700.

**Smallwood Hospital, Redditch.**

The plans of this hospital, the outcome of a charitable bequest of the late Mr. Edwin Smallwood, were prepared by Mr. William Henman, of Birmingham, and the building has been erected by Messrs. G. G. Hains and Sons, Redditch, under the superintendence of Mr. Herbert Lloyd, of Birmingham. The site, building, and furnishing have cost so far, £10,000, and there will be an endowment fund of a similar amount. The building, which is now formally opened, is after the Elizabethan style, and is constructed principally of red brick, with stone gable copings and window dressings. The accommodation for patients consists of a large ward 40 feet by 24 feet at the south end of the wing arranged for eight beds, but capable of holding ten or twelve; a smaller separation ward with two beds, with nurses' room adjoining both wards. The east wing has on the ground floor an out-patient department at the south end, adjoining which are the matron's room, board-room, and nurses' sitting-room. On the first floor, over the board-room, is a children's ward with four beds. The north wing has on the ground floor the kitchen, scullery, &c., and an operating room easily accessible from the wards, also with matron's and surgical store-rooms adjoining. On the first floor are the servants' bed-rooms, with lavatories and bath-rooms for the use of the staff. The main staircase is placed at the angle of the corridors connecting the wings, giving ready access to all parts of the building, and close to a lift which rises from the basement to each floor, adjoining the tradesmen's entrance. The wards are simply but comfortably furnished. The heating is by hot water, and the lighting by gas. Lord Windsor is the first president of the hospital.

